# Systems-thinking approach to identify and assess feasibility of potential interventions to reduce antibiotic use in tilapia farming in Egypt

**DOI:** 10.1016/j.aquaculture.2021.736735

**Published:** 2021-07-15

**Authors:** Andrew P. Desbois, Maria Garza, Mahmoud Eltholth, Yamen M. Hegazy, Ana Mateus, Alexandra Adams, David C. Little, Erling Høg, Chadag Vishnumurthy Mohan, Shimaa E. Ali, Lucy A. Brunton

**Affiliations:** a Institute of Aquaculture, University of Stirling, FK9 4LA, United Kingdom; b Royal Veterinary College, University of London, AL9 7TA, United Kingdom; c Department of Hygiene and Preventive Medicine, Faculty of Veterinary Medicine, Kafrelsheikh University, Kafrelsheikh, Egypt; d Global Academy of Agriculture and Food Security, The Royal (Dick) School of Veterinary Studies, University of Edinburgh, Easter Bush Campus, Midlothian, EH25 9RG, UK; e Department of Animal Medicine, Faculty of Veterinary Medicine, Kafrelsheikh University, Kafrelsheikh, Egypt; f Department of Global Health and Development, London School of Hygiene and Tropical Medicine, United Kingdom; g WorldFish, Penang, Malaysia; h Department of Hydrobiology, National Research Centre, Egypt

**Keywords:** Antibiotic resistance, Antimicrobial resistance, One health, Aquaculture, Tilapia, Egypt

## Abstract

Antibiotics are used in aquaculture to maintain the health and welfare of stocks; however, the emergence and selection of antibiotic resistance in bacteria poses threats to humans, animals and the environment. Mitigation of antibiotic resistance relies on understanding the flow of antibiotics, residues, resistant bacteria and resistance genes through interconnecting systems, so that potential solutions can be identified and issues around their implementation evaluated. Participatory systems-thinking can capture the deep complexity of a system while integrating stakeholder perspectives. In this present study, such an approach was applied to Nile tilapia (*Oreochromis niloticus*) production in the Nile Delta of Egypt, where disease events caused by antibiotic-resistant pathogens have been reported. A system map was co-produced with aquaculture stakeholders at a workshop in May 2018 and used to identify hotspots of antibiotic use, exposure and fate and to describe approaches that would promote fish health and thus reduce antibiotic use. Antibiotics are introduced into the aquaculture system via direct application for example in medicated feed, but residues may also be introduced into the system through agricultural drainage water, which is the primary source of water for most fish farms in Egypt. A follow-up survey of stakeholders assessed the perceived feasibility, advantages and disadvantages of potential interventions. Interventions that respondents felt could be implemented in the short-term to reduce antibiotic usage effectively included: *more frequent water exchanges*, *regular monitoring of culture water quality parameters*, *improved storage conditions for feed*, *use of probiotics* and *greater access to farmer and service providers training programmes*. Other potential interventions included *greater access to suitable and rapid diagnostics*, *high quality feeds*, *improved biosecurity measures* and *genetically-improved fish*, but these solutions were expected to be achieved as long-term goals, with cost being of one of the noted barriers to implementation. Identifying feasible and sustainable interventions that can be taken to reduce antibiotic use, and understanding implementation barriers, are important for addressing antibiotic resistance and ensuring the continued efficacy of antibiotics. This is vital to ensuring the productivity of the tilapia sector in Egypt. The approach taken in the present study provides a means to identify points in the system where the effectiveness of interventions can be evaluated and thus it may be applied to other food production systems to combat the problem of antibiotic resistance.

## Highlights

• A map of the tilapia sector in Egypt was co-produced with stakeholders at a workshop.
• The possible hotspots of antibiotic (mis)use, exposure and fate were identified.
• Possible interventions to reduce antibiotic use were proposed.
• Feasibility and acceptability of interventions were assessed by a post-workshop survey.
• A systems-thinking approach can identify targeted interventions to address antibiotic misuse and antibiotic resistance.

## 1 Introduction

Antibiotic resistance (ABR) is one of the greatest challenges we face in the 21st century and it is a classical One Health problem with human, animal and environmental components (Robinson et al., 2016). Antibiotic-resistant bacteria and antibiotic-resistance genes (ARG) transfer between these components, which complicates the tracking of their flow and adds complexity when developing solutions (Robinson et al., 2016). The problem of ABR must be addressed to maintain the effectiveness of antibiotics in food production and for patients in healthcare settings.

The global aquaculture sector is a major user of antibiotics, where these agents are applied to maintain the health and welfare of stocks, though usage and practices vary widely across the world (Henriksson et al., 2018; Lulijwa et al., 2020). Accordingly, the problems posed by ABR vary, though much of the burden of the issues encountered falls on low- and middle-income countries (LMICs), where controls on antibiotics may not be as strict or as strongly enforced as in wealthier counterparts (Robinson et al., 2016; Henriksson et al., 2018).

ABR in aquaculture poses a threat to human health and to the contamination of the environment with antibiotic residues and resistant organisms. Many of the classes of antibiotics used in aquaculture are identical to those used to treat terrestrial farm animals and human patients (Rico et al., 2013; Done et al., 2015; Lulijwa et al., 2020). In LMICs, aquaculture systems are highly complex and often integrated with other food production systems (Cantas et al., 2013; Chuah et al., 2016; Watts et al., 2017; Shen et al., 2019), such as through sharing of common water sources, making them highly vulnerable to the introduction and widespread dissemination of ABR (Cabello et al., 2016; Brunton et al., 2019). The complexity of these systems creates multiple points for human exposure to antibiotic residues, ARGs and resistant organisms; our earlier study identified three key pathways for human exposure to these to occur through occupational duties, consumption of contaminated food and environmental exposure (Brunton et al., 2019).

Egypt produces more fish from aquaculture than the rest of Africa combined, with production trebling since 2005 to 1.56 million tonnes in 2018 (FAO, 2020). Aquaculture in Egypt is dominated by the production of Nile tilapia (*Oreochromis niloticus*) in earthen ponds, with production concentrated in the northern regions of the Nile Delta. The sector has undergone considerable expansion in recent decades, driven by the development of privately-owned hatcheries and feed mills (El-Sayed, 2017), and Egypt produced 1,051,444 t of tilapia in 2018 (FAO, 2021b). The sector is composed mainly of small-scale producers and the value chain, including production constraints and consumer behaviour and preferences, has been described in detail recently (Eltholth et al., 2015). Like tilapia producers elsewhere, fish farmers in Egypt experience disease challenges (Ali et al., 2020), including infectious diseases caused by bacteria such as *Aeromonas hydrophila*, enterococci species, *Pseudomonas fluorescens* and *Streptococcus iniae* (Aly, 2013; Osman et al., 2016; Osman et al., 2017). Antibiotics are used by farmers to treat infections in their stocks but antibiotic-resistant pathogens have been reported to cause infections in farmed tilapia in Egypt (Eltholth et al., 2015; Ishida et al., 2010; Osman et al., 2016; Osman et al., 2017). It is essential that this problem is addressed to ensure the sustainability and continued productivity of the sector, which supports livelihoods, particularly in rural communities, and is a major source of affordable animal protein for the country's population. Indeed, almost all tilapia production in Egypt is for domestic consumption and only 2059 t of product were exported in 2018 (FAO, 2021a).

Many approaches can help to mitigate against the problems posed by ABR and this includes interventions that decrease overall use of antibiotics, such as by reducing need and dependency on them. When identifying mitigation measures for ABR, including ways to reduce the need for their application, it is vital to include all relevant stakeholders in the process and the decision-making. Such participatory approaches capture the range of views, lead to a sense of collective responsibility towards the problem, and ensure a feeling of shared ownership towards solutions (Evans and Terrey, 2016; Blomkamp, 2018). Systems thinking attempts to integrate the multi-level aspects of a system, including actors, processes, and governance structures. The approach can be used to generate a map that can then be used to understand how an intervention in one part of the system to address a problem will impact on the functioning of the entire system, thus permitting the recognition of consequences in an attempt to avoid unintended negative outcomes elsewhere in the system (Peters, 2014). Brunton et al. (2019) used a participatory system-thinking approach to consider points in aquaculture systems in Vietnam where ABR could emerge, or be enriched through selection, by exposure to antibiotics and other selectors of ARGs.

In this present study, we aimed to identify potential interventions that may reduce antibiotic use (ABU) in tilapia production systems in Egypt through a participatory systems-thinking approach with key stakeholders. To meet this aim, it was necessary to (1) map the tilapia production system in Egypt; (2) identify hotspots of ABU, exposure and fate within the system; (3) describe approaches that would promote fish health, including the use of alternatives to antibiotics, and (4) conduct a follow-up survey of key stakeholders to assess the perceived feasibility, advantages and disadvantages of potential interventions to reduce or prevent ABU, including actual or perceived barriers to implementation.

## 2 Methods

### 2.1 Stakeholder workshop

A one-day participatory workshop was organised with aquaculture stakeholders in Kafrelsheikh, Egypt, on 10 May 2018. The workshop involved 44 participants from a range of disciplines and stakeholder groups, including professionals from the aquaculture, livestock and veterinary sectors working in the private sector (e.g., tilapia producers, input producers, agrovets, feed producers, and pharmaceuticals), academics, and employees of international development and public sector institutions (Table 1). The expertise amongst participants included aquaculture and aquatic health management, veterinary epidemiology and public health, food safety, microbiology, marine biotechnology and medical anthropology. This range of participants was invited to capture a diversity of perspectives, expertise and experiences, and to maximise engagement and collaboration amongst distinct stakeholder groups. Participants were allocated to three groups, each containing a mix of expertise. Outputs were compared between the groups, so to permit cross-checking and discussion of the outputs as a method of validation. Each group contained two members of the research team, one of whom facilitated the activity (the facilitator) and another who took notes of discussions throughout the process (the recorder). All workshop activities were facilitated in English and simultaneously translated into Arabic by members of the research team and participants, where required.

**Table 1:** Professions of workshop participants. Some participants represented more than one organisation.

Organisation	n
UK-based academics (from 3 institutions) *(research team)*	8
Egypt-based academics (from 4 institutions)	13
WorldFish Egypt	5
National Research Institute (ICLAR)	1
Feed factory owner	1
Farmers/fish farm owners	15
Pharmaceutical company representative/trader	1
General Organisation of Veterinary Services	2

The workshop activities consisted of introductory presentations, the main mapping exercise, and a final plenary activity. During the introduction, participants were provided with a contextual overview of tilapia production and value chain in Egypt, including post-harvest operations, the workshop aim and objectives and planned activities, as well as an introduction to systems thinking and its application to food production systems. To familiarise participants with the systems concept and the mapping process, an exercise was conducted whereby each group was asked to draw a simple system on large whiteboards or paper sheets affixed to the wall, exemplifying ‘making a cup of tea’. Groups had to identify elements of the system such as tangible and intangible components, including actors, infrastructure, governance systems and relationships, and economic and environmental factors. The exercise introduced participants to the mapping process and the importance of establishing boundaries (i.e., ‘edges’) with other systems. This approach is based on the methodological framework proposed by the Network for the Evaluation of One Health (NEOH) (Rüegg et al., 2018).

### 2.2 Mapping of the tilapia system

**Fig. 1:**
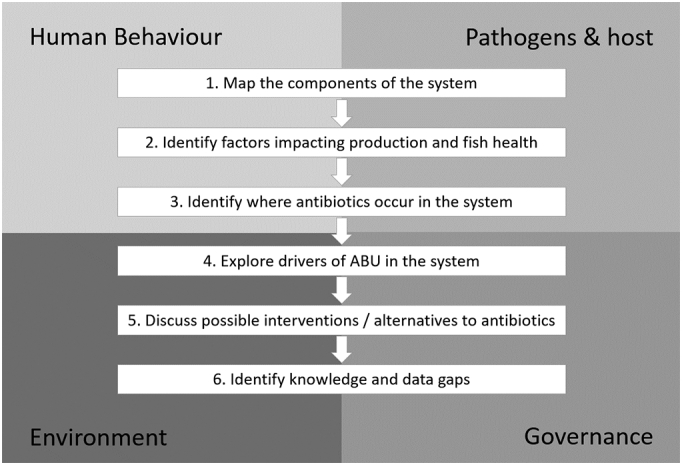
Workshop objectives of the mapping activity, framed within four dimensions. Participants were asked to consider each of the dimensions when working through each objective.

Each group was guided through the mapping activity to accomplish a series of objectives, and these objectives were framed to consider four dimensions of the system (Fig. 1). The groups were asked to:

• *Map* the components of the system (e.g. production stages, inputs, outputs, actors and relationships);
• *Identify* any factors in the system that impact production and fish health;
• *Identify* where ABU and antibiotic residues occur in the system;
• *Explore* drivers of ABU in the system and decision making processes of stakeholders;
• *Discuss* possible interventions and alternatives to antibiotics, and
• *Identify* knowledge and data gaps.

The four dimensions within which these objectives were considered were the *human dimension*, related to stakeholder behaviour; *the pathogen and host factors*, including information on common pathogens and diseases in tilapia; *the environment*, considering information on related systems such as the agricultural and drainage systems; and *governance*, considering the influence and power of different stakeholders on decision-making or practices. A protocol was developed by the research team members to facilitate the mapping process, which included the key elements to be captured, such as suggestions for alternatives to antibiotics, and prompting questions to promote discussion and participation.

Participants were asked to identify hotspots on the maps where antibiotic residues or ARGs may be present. Hotspots were defined in three ways, concerning system *nodes*, system *inputs* and *outputs*, and system *management*. Nodes in the system represent junctions, where there is active use of antibiotics, or where there is a potential presence of antibiotic residues or ARGs due to the flow of products through the system (e.g., hatchery, nursery ponds, grow-out ponds, poultry farms, etc.). Products, inputs or outputs of the system could contain antibiotic residues or select and/or enrich for ARGs (e.g., antibiotics, feed, manure from poultry farms, drainage water, etc.). Management practices may involve the active use of antibiotics or use of products containing antibiotic residues or ARGs (e.g., during the fertilisation stage of pond preparation using poultry manure, the crop production using drainage water from aquaculture farms, etc.).

### 2.3 Plenary activity and follow up survey

The main findings from each group were summarised and presented back to all participants in a plenary session, highlighting the key components of the system, major factors impeding production and good management, drivers of ABU, the flow of antibiotics and residues in the system, and areas of disagreement or inconsistency between the groups. Following this, a concluding exercise was conducted to compile information on alternatives to antibiotics that had emerged during the mapping process in each group. In addition, participants were asked to suggest any additional alternatives to antibiotics and interventions that may reduce ABU by improving fish health and to rank these based on perceived feasibility and effectiveness.

These discussions provided the foundation for a post-workshop survey to investigate the perceptions and attitudes of the workshop participants, and additional stakeholders not in attendance, towards alternatives to antibiotics and interventions aiming to improve fish health management practices and thus reduce ABU (Appendix A Supplementary material). The survey was structured and developed by the research team using information gathered at the workshop, a review of existing literature, and expertise within the team. This resulted in a list of 31 alternatives to antibiotics, or potential interventions, which were divided into eight groups. The groups were: *farm management practices*; *new breeds and genetic improvement*; *disease diagnostics*; *monitoring and control*; *biosecurity*; *rewards and incentives*; *education and training*; and *alternative therapies and products*, e.g. vaccines, immunostimulants and others. Survey respondents were asked about the perceived *feasibility* (short term, long term, or not possible to achieve), and *advantages and disadvantages* (affordability, availability, ease and speed of application or implementation, and effectiveness), and then to rank the interventions in terms of overall perceived importance to reduce ABU. The survey was translated into Arabic and purposefully distributed by email to workshop participants via SurveyMonkey, and in-person through the networks of the research team in the Kafrelsheikh Governorate (completed either by email or face-to-face), aiming to reach aquaculture professionals, particularly farmers.

### 2.4 Analysis of maps, consensus map and validation

At the end of the workshop, each map developed by the three groups was photographed and translated by the research team into a single, consensus digital diagram using the web-based Lucidchart software (https://www.lucidchart.com/). The components of the consensus map were classified broadly into: (1) *pre-harvest activities and production*; (2) *inputs*, such as feed and chemicals; (3) *waste products*; (4) *post-harvest activities* and (5) *governance, roles and responsibilities of actors* taking decisions in the systems particularly in regard to fish and public health.

## 3 Results

### 3.1 Mapping tilapia production systems

#### 3.1.1 Description of the system

**Fig. 2:**
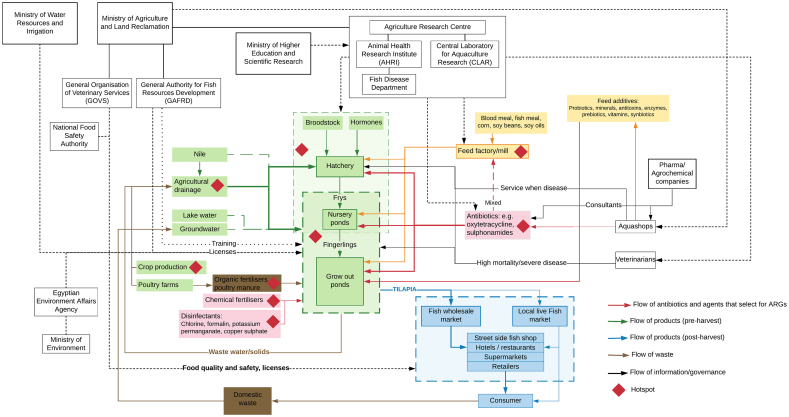
Consensus map of the farmed tilapia production system in Egypt.

Three workshop groups each constructed a map of the tilapia production system in the Nile Delta and it was apparent that each group had focused to varying degrees on distinct parts of the system, which probably reflected the different experiences of the participants in the groups. A consensus system map was constructed from these original hand-drawn maps (Fig. 2), and this was relatively simple given the near complete absence of contradictory information. Tilapia is cultured almost exclusively in earthen grow-out ponds that are prepared initially by draining and removing sediment. The pond is then sun-dried and disinfected with lime, before filling with water. Thereafter, a bloom of microorganisms, most likely phytoplankton, zooplankton and bacteria, is initiated in the pond water through fertilisation with organic fertilisers such as poultry manure. This microbial community formed in the pond improves the water quality, provides additional nourishment to the fish, and may confer some protection against pathogens through niche closure and stimulation of the host immune system (Ahmad et al., 2017).

Grow-out earthen ponds (mean area ca. 1.3 ha) are usually located in the northern regions of the Nile Delta, with a main water supply of agricultural drainage water. Grow-out ponds are stocked either with mono-sex fry obtained directly from the local hatcheries, or by fingerlings that have been over-wintered in on-farm nursery ponds. The stocking of grow-out ponds usually starts in March. Almost all tilapia farmers cultivate tilapia in polycultures with two species of mullet, the flathead grey mullet (*Mugil cephalus*) and thin lip mullet (*Liza ramada*). Over the last 10 years, there has been a shift from mostly extensive production to semi-intensive and intensive systems due to the increased availability of hatcheries and commercial feed. Very few farmers have access to electricity and a limited number of farms use some type of aeration; most farmers use diesel pumps to pump water in and out of ponds. Feed mills provide feed formulated to contain a variety of raw ingredients, including soy bean meal (and oil extracted from the beans), corn, and blood and fish meal. More than 90% of fish farms are using formulated commercial feed and almost all farmers add feed to the pond manually. Some feeds are supplemented with additives, such as probiotics, prebiotics, minerals, enzymes and antimycotoxins, which aim to prolong the shelf life, increase digestibility and enhance the fish health status. These supplements may be added during manufacture at the mill, but often are incorporated into the feed by farmers themselves. Supplements are sourced from specialist retailers called ‘aqua shops’ marketing products exclusively for fish producers, although these outlets may also sell products intended for use in other livestock.

The water entering the system is derived mainly from agricultural drainage from terrestrial crop production (ca. 70% of farms), with a small proportion of farms deriving water from the Nile (ca. 20%) and groundwater (ca. 10%). A small number of ponds have access to irrigation water, but this is illegal to use for fish production, while some others have access to lake water. The water is channelled via a network of interconnecting canals that link the fish farms. Few farms have access to groundwater to fill or top up ponds during water exchange meaning most farms rely on the canals, but this water may contain chemicals, sediment and microorganisms. All farms release wastewater into the canal network, which exacerbates potential cross-contamination and means water of poor quality, containing potentially pathogenic microorganisms and drug residues, may be introduced back into the tilapia ponds.

Fish exhibiting suspect clinical signs of infectious disease (e.g., bacterium or parasite) may be treated by adding disinfectants to the pond water, such as formalin, copper sulphate or potassium permanganate. During episodes of increased morbidity and mortality in the grow-out ponds, farmers often reduce the water level in the pond before adding medications to the water. However, low diagnostic capacity, stemming from a lack of trained veterinarians with expertise in aquatic animal health, and reliance on a range of providers of varying quality for diagnostic support, means that diseases are likely to be commonly misdiagnosed. In addition, antibiotics are available over the counter (i.e. without the need of a veterinary prescription), from aqua shops and similar retailers, where they may be incorporated into feed, either by the feed mill upon request or at the farms by the farmers. Opinions of whether feed mills add antibiotics to feed or not were inconsistent across workshop groups. One group suggested this to be a common practice, while another group maintained that feed mills do not add antibiotics to feed. However, further discussions with a feed mill owner confirmed that feed mills did add medications to feeds upon request, but this was rare and costly. Furthermore, antibiotics may be added to the culture water to treat diseased fish at hatcheries and nurseries.

At harvest, fish are netted and transported to the markets without refrigeration or via a rudimentary cold storage system (i.e. ice), which means the fish must be sold within a few hours (Fig. 2, blue section). A limited network of wholesalers supplies a range of sales outlets, including supermarkets, restaurants, fry shops, and street side vendors.

The workshop maps showed that various government ministries are involved in governing the tilapia production system. Ministerial involvement aims to promote and expand the industry. *The Ministry of Agriculture and Land Reclamation* governs three main authorities: *The General Authority for Fish Resources Development* (GAFRD), *The General Organisation for Veterinary Services* (GOVS) and *The Agriculture Research Centre* (ARC). The GAFRD drafts the legislation and regulations for fisheries and manages farm licensing, aquaculture land use regulations, as well as extension and research services. The GOVS controls the import/export of fish and conducts aquatic health surveillance, though this is not yet in place. The ARC includes *The Animal Health Research Institute* (AHRI), which contains a department focused on the management of fish diseases, and the *Central Laboratory for Aquaculture Research* (CLAR). *The Ministry of Water Resources and Irrigation* controls water use and authorises the issuing of licences by *The Ministry of Agriculture and Land Reclamation* to farmers to permit them to take water from the agricultural drainage canals, though farmers rarely obtain such permits. It is illegal to take water from the Nile for aquaculture use, although this may occur to a small degree.

*The Ministry of Environment* oversees the use of land for aquaculture purposes through the Egyptian Environment Affairs Agency (EEAA). The establishment of new fish ponds must be subject to an Environmental Impact Assessment (EIA), which is reviewed by the EEAA. The scope and depth of the EIA is determined by the EEAA on the basis of the information provided in the application, and will vary depending on factors such as whether the farm is situated in an urban setting, or an ecologically sensitive or protected area.

*The National Food Safety Authority* (NFSA) carries out inspections and performs laboratory analysis of fish samples for the domestic market and to meet export conditions as required. *The Ministry of Higher Education and Scientific Research* includes educational and research institutes that support the tilapia sector with qualified aquaculture specialists and research outputs to improve the industry.

Boundaries to the tilapia production system map include: the sources of ingredients for feed, including feed additives; international and overseas agencies of governance; crop production; poultry (and other livestock) production; production of chemical inputs (e.g., disinfectants, antibiotics); feed supplements; ice, energy, and brood stock; domestic wastes that may contaminate water sources; production of equipment such as nets and tanks; sales and marketing outlets; and service providers (e.g. veterinarians etc.).

#### 3.1.2 Factors that impact production and fish health

Different stakeholders participating in the workshop emphasised factors that impact production and fish health. Producers mentioned several factors that often appeared to be interconnected, including the high prices of the inputs and dependency on value chain stakeholders, fluctuation of market prices of the products, access to clean water and irrigation systems, losses due to diseases, and regulations such as the restrictions in the irrigation systems and the marketisation of products.

Fish health and management stakeholders mentioned occasional moderate mortality rates and outbreaks, but disease was often described to be linked to management deficiencies. In particular, poor water quality was described to be a key factor impacting fish health by different stakeholders. Further, workshop participants observed that pressure to produce a timely harvest favoured short-term solutions and prophylactic use of veterinary products, instead of investing or focusing on improved disease prevention and control, management and biosecurity.

Stakeholders mentioned the generalised lack of monitoring for water quality parameters at the farm level, and many producers rely solely on organoleptic characteristics to determine water quality. Similarly, there is no widespread use of disease diagnostic tests and antibiotic susceptibility testing, which often results in recurrence of infectious diseases and ineffectual empirical treatments.

#### 3.1.3 Antibiotics in the system

Fish mortality was the main driver directly leading to ABU, generally described to be applied to treat stocks with evidence of clinical signs, but participants also mentioned occasional prophylaxis when neighbouring farmers were experiencing an outbreak suspected to be due to an infectious disease. The most common product referenced was oxytetracycline (a tetracycline), though sulphonamides and other antibiotic substances were also mentioned in discussions. During a visit to two aqua shops in the locality of the workshop by the research team, products containing dihydrostreptomycin (an aminoglycoside), oxytetracycline, ciprofloxacin (a fluoroquinolone), erythromycin (a macrolide), and sulphadimidine (a sulphonamide) were observed for sale.

One direct way antibiotics enter the system is from the aqua shops and similar retailers. Antibiotics are used to treat diseases and these may be added to feed or to the pond water particularly where water-body volumes are smaller such as at hatcheries and nurseries, though grow-out pond water levels can be reduced for treatment in this way by releasing water into the agricultural drainage canals. Antibiotics may also be introduced into the tilapia production system as residues in wastewater derived from other systems, including domestic waste, which enters into the agricultural drainage canals used subsequently by fish farmers to replenish the ponds. Thus, these canals represent a potential source and reservoir of antibiotic residues, which allows them to be transported widely across the system. A further possible source of antibiotic residues and resistant bacteria entering the tilapia production system may be the use of organic wastes, such as untreated poultry manure, to fertilise the ponds prior to stocking.

Having plotted the movements of antibiotics within the system, the workshop groups proceeded to identify hotspots where the presence of antibiotics or residues, or other conditions or compounds, may select for resistant organisms containing ARGs. Hotspots identified included: *hatcheries and nurseries*, where antibiotics may be applied to prevent or treat disease events; *the drainage canals* and the water contained within, where residues from various systems are transported and may accumulate; and the *grow-out ponds* at the farms, where antibiotics may be introduced during fertilisation with poultry manure, during application of medicated feeds, from the introduction of contaminated canal water, or where the application of co-selectors of ARGs such as disinfectants are applied. Further hotspots included the *feed mills*, where antibiotics may be incorporated into fish feed, and in *crop and poultry production*, where antibiotics may be applied to prevent or mitigate infectious diseases or as growth promotors.

### 3.2 Attitudes and perceptions of survey respondents

**Fig. 3:**
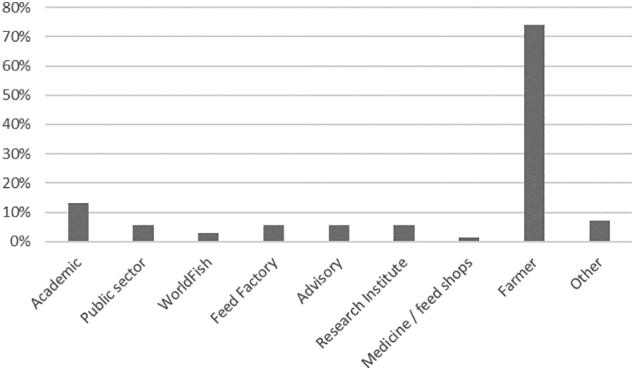
Occupations of respondents to the post-workshop survey.

**Fig. 4:**
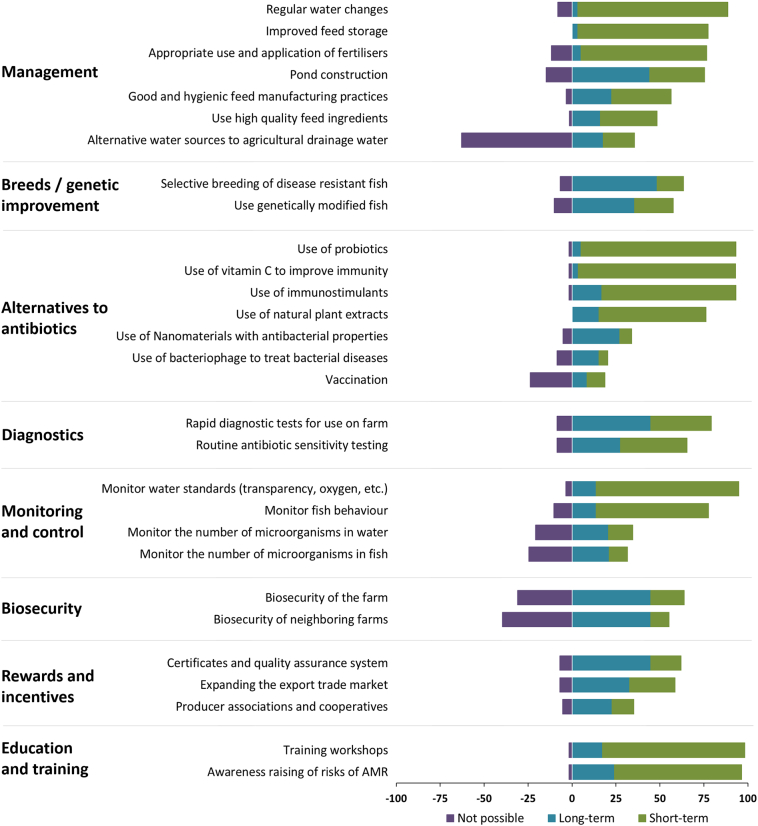

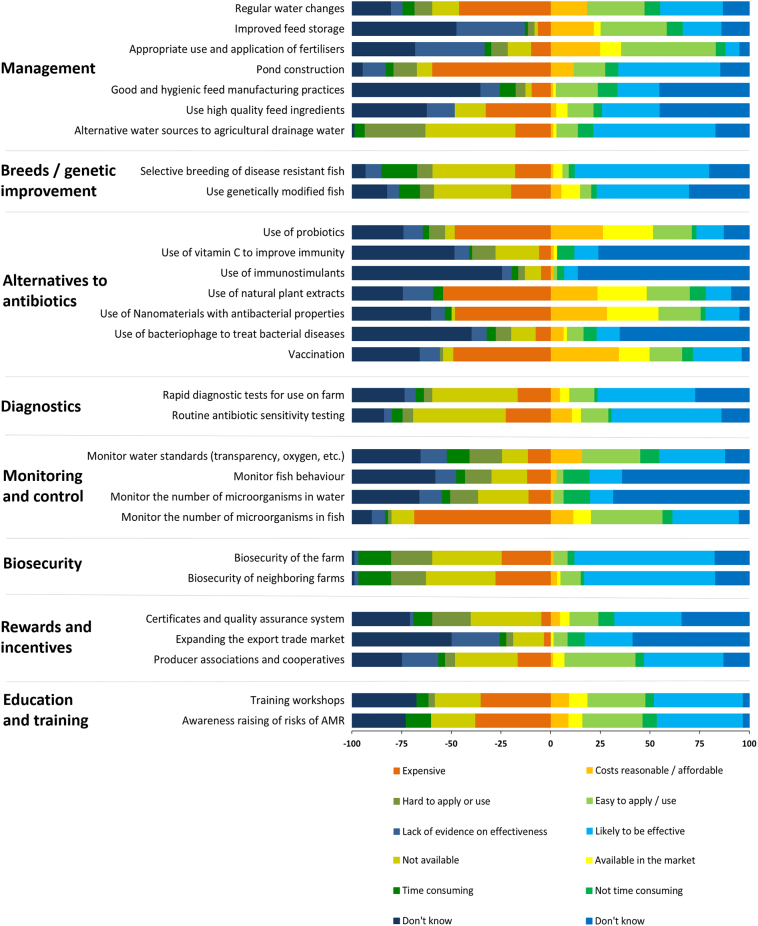
a: Summary of responses to the post-workshop survey on the feasibility of various interventions to reduce ABU and improve fish health. b: Summary of responses to the post-workshop survey on the advantages and disadvantages of various interventions to reduce ABU and improve fish health.

A total of 69 responses to the survey were received within six weeks of the workshop. Respondents represented a variety of stakeholders as described in Fig. 3, but primarily fish farmers (*n* = 51). Of the 69 respondents to the survey, 74% were not present at the workshop. Response rate for individual questions ranged from 84% to 100%, with 84% of respondents completing the survey in full. All 51 farmers were surveyed face-to-face and completed the survey in full. A summary of the responses from the entire survey is presented in Fig. 4a and b.

Improving water exchange practices and storing feed in more suitable conditions were perceived to be most achievable in the short-term to improve fish health, mainly because these actions were felt to be effective and relatively easy and inexpensive to implement. Using pond fertilisers most appropriately was perceived to be a possible solution to be implemented in the short-term, but only 10% of respondents thought that this approach would actually be effective in improving fish production and reducing ABU. There was an agreement that pond modifications allowing fish to select thermal preference (Cerqueira et al., 2016; Tabin et al., 2018) could be achieved and such changes were felt strongly to be effective, but there was a split in opinion on the possible timescale required for this intervention, which was also perceived to be expensive. More than 40% did not know whether better quality, nutritious feed than what is currently available could be achieved (e.g., by sourcing higher quality ingredients and improved manufacturing practices) and this likely reflects the lack of knowledge amongst respondents regarding the potential impact on productivity and fish health, or what is needed for this to be accomplished. Furthermore, it was clear that alternative sources to the agricultural drainage water were not thought to be possible due to the current restrictions on water use imposed by existing legislation, despite being identified to be an effective way to improve fish production and reduce ABU.

There was strong support for the development of genetically improved fish for disease resistance as a long-term goal and an effective strategy to reduce disease and ABU, but disease-resistant strains were recognised to be unavailable and costly to develop. There was strong support for the introduction of rapid diagnostics and a role for the routine application of antibiotic sensitivity testing as effective ways to reduce disease and ABU, although there was a split in opinion on whether this was achievable. The majority of respondents indicated that such tests were unavailable, with others saying the prohibiting factor in their implementation was high cost.

Respondents perceived short-term benefits to be delivered by monitoring water quality parameters, such as dissolved oxygen levels and water ‘transparency’, and making an inventory of fish clinical signs and behaviour to inform diagnosis, which largely stemmed from being relatively easy to implement, although there were concerns around the cost of tests. Improved biosecurity was perceived to be achievable by most respondents as a way to reduce disease outbreaks and ABU, with most thinking this would be effective. Although almost a third of respondents felt this would not be possible due to the cost, and the difficulty and time needed to achieve improvements.

There was support for developing export markets, certification and quality assurance schemes as long-term, effective strategies to reduce disease outbreaks and ABU. However, many respondents were unsure of the benefits and barriers to developing export markets. Producer associations and cooperatives were thought by many respondents to be easy to establish, though many respondents were unsure whether this could be realised. The questions pertaining to education and training showed an appetite amongst respondents for learning and acquiring new skills, and this was seen as achievable in the short term, easy and effective for reducing ABU and disease outbreaks, although concerns were raised about cost (mostly) and a lack of availability.

Various alternatives to antibiotics for preventing and treating disease had been proposed during the workshop discussions. Amongst these, the use of vitamin C, probiotics and immunostimulants all received strong support and were perceived to be capable of delivering benefits in the short-term due to their effectiveness, low cost, ease of availability and ease of use. Application of natural plant extracts was perceived to offer benefits for similar reasons; however, their use was envisaged further in the future. Few respondents viewed vaccination positively in its potential effectiveness to reduce disease and ABU, possibly due to lack of availability, and there was little knowledge of the potential of bacteriophage therapy in disease control.

**Fig. 5:**
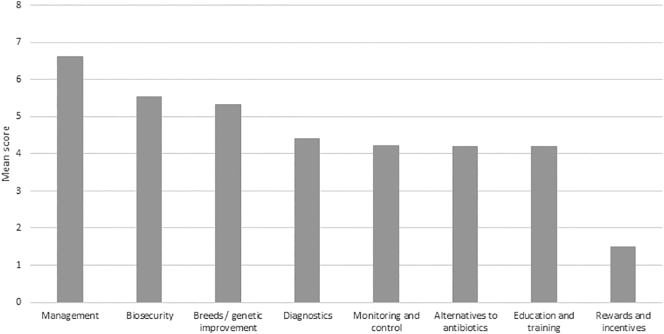
Mean score of importance for each group of interventions (8 = highest score available and 1 = lowest score available).

Finally, in a ranking exercise, improved farm management practices and biosecurity were ranked highest in terms of importance to reduce ABU and improve aquaculture, with (presumably overt) rewards and incentives perceived to be least important by a considerable margin (Fig. 5).

## 4 Discussion

The aim of this study was to identify potential interventions that may reduce ABU in farmed tilapia production in Egypt using a participatory systems-thinking approach with key stakeholders from this industry. A consensus map of the farmed tilapia production system was created and used to identify possible hotspots of ABU and emergence of ABR, routes of antibiotic flow through the system, and interactions with other systems. Thereafter, approaches that would promote fish health and reduce or prevent ABU were proposed, discussed and their perceived feasibility assessed by key stakeholders through a survey that followed the workshop.

Previous assessments of the tilapia sector in Egypt using value-chain analysis have provided information concerning structure and performance, and identified key constraints within the sector (Macfadyen et al., 2012; Eltholth et al., 2015). Here, a systems-thinking approach was taken to integrate the multi-level aspects of the system, such as governance and the environment, and to provide a framework for exploring how a proposed intervention to mitigate ABR at one point in the system might affect the functioning of the entire system. Such an approach is needed due to the complexity of the system, and the participatory nature of the approach is beneficial because it can capture a range of views, leads to a sense of collective responsibility towards a problem, and provides a feeling of shared ownership towards solutions (Evans and Terrey, 2016; Blomkamp, 2018). Mapping the system provided the means to visualise ABU in the sector, which allowed for identification of how and where in the system antibiotics are introduced, how and where they are transmitted, and the points in the system that represent potential hotspots for the emergence and selection of ABR.

In a previous study, a similar approach was used to identify hotspots for the emergence of ABR in striped catfish and shrimp farming in Vietnam (Brunton et al., 2019). Similarities between the aquaculture production systems in Egypt and Vietnam emerged with respect to the locations of hotspots for the emergence of ABR, with culture ponds, feed mills and markets identified in both exercises (Brunton et al., 2019). Moreover, these hotspots hold the potential for human exposure to antibiotic residues, ARGs and antibiotic-resistant bacteria. Other studies have reported similar findings, with a variety of antibiotic residues being detected in aquaculture ponds in Vietnam (Le et al., 2005; Andrieu et al., 2015; Giang et al., 2015; Nakayama et al., 2017), and antibiotic-resistant bacteria detected in aquaculture ponds in Egypt (Ishida et al., 2010; El-Gohary et al., 2020).

Mapping the farmed tilapia production system highlighted the interaction between aquaculture and agricultural systems, with water contaminated with antibiotic residues able to permeate the whole delta and move between other systems, including crop and terrestrial animal production systems. For example, poultry manure and litter are commonly used as fertiliser for fish farming due to its nutritional value (Mahmoud and Abdel-Mohsein, 2019), but it is rarely treated (e.g. pasteurised) before use (Eltholth et al., 2015). This can represent a possible source of antibiotic residues (Petersen et al., 2002), in particular tetracyclines, which are used extensively in poultry production in Egypt (Mahmoud and Abdel-Mohsein, 2019). Tetracycline concentrations exceeding European Union maximum residue limits (100 μg/kg) have been detected in wild and farmed tilapia in the Nile delta region, with greater concentrations in farmed versus wild samples suggested to be due to the use of contaminated poultry manure as pond fertiliser (Mahmoud and Abdel-Mohsein, 2019). Indeed, high concentrations of tetracycline residues were detected even on fish farms with no recent history of using this antibiotic (Mahmoud and Abdel-Mohsein, 2019), indicating soil and groundwater may act as reservoirs for antibiotic residues (Boxall et al., 2003). In addition, high levels of ARGs have been detected in poultry manure and litter, and these genes may be carried by pathogens (Abu-Elala et al., 2015; Mahmoud and Abdel-Mohsein, 2019) and commensal bacteria alike.

Availability of and accessibility to irrigation canal water was a key challenge that emerged during both the workshop discussions and from the survey data. Agricultural drainage water is the main water supply for most fish farms in this region of Egypt, but it represents a major biosecurity risk and is a key potential source of antibiotic residues, ARGs and antibiotic-resistant bacteria that may contaminate the culture ponds (Eltholth et al., 2015). The use of irrigation water in fish ponds is not allowed because it is prioritised for agricultural use (GAFRD Law No 124/1983). Since agricultural drainage water can be detrimental to fish health and growth, it has been suggested instead to use irrigation water primarily in aquaculture and then use the aquaculture effluent in agriculture as a source of water and organic fertiliser (Henriksson et al., 2017). While this would not stop chemical and biological hazards from fish ponds being disseminated to the wider environment, it might lead to an overall reduction in the transmission of such hazards, if water quality improvements, including lower levels of pathogens, lead to reduced application of antibiotics in aquaculture. This is worthy of consideration but may not be possible in Egypt, as the land set aside for aquaculture is mostly downstream of agricultural crops. According to workshop participants, about 20 to 30% of water is exchanged every day under normal circumstances; however, the frequency and volume of water exchanged depend on the stage of production, size of fish and the season (Eltholth et al., 2015). The main purpose of pumping water is to increase the dissolved oxygen level in pond water. During outbreaks, some farmers reduce the level of pond water to the minimum to administer a treatment to the fish (Eltholth et al., 2015). Many respondents to the survey reported that more frequent water exchanges would be feasible in the short term, and felt that this would be effective in improving fish health. This may not be the case where the quality of incoming water is inadequate, and thus may be counter-productive, which could indicate some misunderstanding amongst respondents of the water quality problems and how best to solve them.

Demand for fish is increasing in Egypt due to a growing population and increased per capita consumption in the last two decades, likely due to economically incentivised changes in consumer preferences and increased accessibility (i.e., low-cost domestic fish production, improvements in distribution networks, etc.) (Murphy et al., 2020). Indirect price controls on tilapia have been imposed by the government through the introduction of an export tax for tilapia, and this policy has created tension between a government aiming to ensure a reliable, low cost and secure supply of animal protein for the population in the country and tilapia producers keen to maximise profits. Survey respondents expressed a strong desire to explore and expand exporting opportunities, and it was suggested that this could act as a driver to reduce ABU by raising production standards and allowing farmers to sell fish at higher prices, as observed elsewhere (Nguyen and Jolly, 2020). Currently, a notable barrier to exporting tilapia from Egypt is the need for a comprehensive residue monitoring system and disease testing framework, both of which are required for export to the European Union and the United States (Fitzsimmons, 2008; Eltholth et al., 2015). In addition, the costs associated with higher standards of production, limited processing facilities and ability to add value, and lack of by-product industries represent important barriers to export (Fitzsimmons, 2008; Eltholth et al., 2015). Previous research has shown the importance of the local market, where tilapia consumption is greater amongst the communities in high production areas than in non-production areas where fish may be available only once per week on market day (Eltholth et al., 2015). Currently the tilapia market is dominated by a few large wholesalers who control the price (Soliman and Yacout, 2016), and the national government seeks to ensure the tilapia industry offers food security for the population, meaning production for the domestic market is prioritised. Indeed, the building of government aquaculture farms, such as the Birkat Ghalioun fish pond project (Feidi, 2018), illustrates the government's plans to meet the increased demand for fish for the population (El-Gohary et al., 2020). However, it is conceivable that greater revenues resulting from exporting tilapia could provide the income necessary to secure the supply of alternative high-protein foodstuffs.

Improvements to diagnostic capacity and a need for alternatives to antibiotics were frequently mentioned at the workshop and these deficiencies are often highlighted by aquaculture farmers in LMICs (Ninawe et al., 2017; Stentiford et al., 2017; Garza et al., 2019). While improved diagnostics were considered favourably by respondents, the consensus was that cost was a barrier and it is difficult to envisage how this situation will improve to meet the needs of farmers in the near future, given the significant investment that would be necessary. The use of probiotics and immunostimulants may offer a feasible and relatively inexpensive option to reduce the need for antibiotics by improving the health and welfare of stocks (Chauhan and Singh, 2019). There have been numerous trials with these products in tilapia, for example dietary supplementation of Nile tilapia with *Bacillus subtilis* and *Saccharomyces cerevisiae* was associated with improvements in selected measures of immunity and an enhancement of fish flesh quality (Elsabagh et al., 2018; Opiyo et al., 2019). In addition, probiotics can be used to modify the microbial composition of pond water to improve water quality (De Schryver et al., 2014). Still, much more needs to be done to ensure probiotics are tested appropriately and confirmed to be effective under farm conditions (Partridge, 2016; Knipe et al., 2020).

Meanwhile, few survey respondents viewed vaccination positively for its potential effectiveness to reduce disease and ABU, even though vaccination programmes can lead to massive reductions in ABU, such as has been seen in Atlantic salmon farming around Scotland and Norway (Sommerset et al., 2005; Adams, 2019). It was discussed at the workshop that there are few vaccines available for tilapia, and that these are currently injection vaccines, whereas immersion vaccination would be more appropriate. Although there was little knowledge of the potential of bacteriophage in disease control, this is less surprising given it is not an established technology like vaccination (Gon Choudhury et al., 2017). The lack of appreciation for the potential of vaccination programmes to improve fish health may be due, in part, to a lack of awareness and lack of availability of vaccines for tilapia diseases. However, it is encouraging that there was an appreciation by survey respondents for the importance of education and training to improve the ABR situation in tilapia farming, indicating a possible avenue to raising attention to the success of vaccination programmes in improving fish health and reducing ABU. More broadly, this appetite for learning provides a platform that can be used to improve many aspects of tilapia production in the country. Indeed, the impact of training farmers in best management practices (BMP) has been assessed in the aquaculture sector in Egypt (Dickson et al., 2016; Henriksson et al., 2017). Providing farmers with BMP training improves farm profitability though not necessarily productivity, and the considerable economic, social and environmental gains from investment in training of fish farmers in Egypt is well recognised (Dickson et al., 2016). Indeed, Henriksson et al. (2017) used lifecycle assessment to show that BMP training of tilapia farmers in Egypt reduced lifecycle environmental impacts by 22%.

A limitation of this present study was the absence of policy makers from government agencies, as this would have provided broader context and a better understanding of the decision-making processes at national level. However, the presence of government representatives could have altered the dynamic of the workshop, and influenced the responses of the ground-level stakeholders identified for inclusion in this present study. It was important to capture the views of these stakeholders on the acceptability and feasibility of the interventions, given that the proposed interventions will have greatest impact upon them and will most likely need to be implemented by them. Having captured these views, engagement with government is a priority and is essential to understand how best to influence policy, particularly on water use and the constraints to exporting tilapia to different territories, and how any barriers may be surmounted. In addition, this engagement would be useful for identifying potential resources and means to deliver the desired increase in diagnostic capacity. Therefore, a smaller, focused follow-up workshop is proposed to include a broader range of stakeholders, including those representing relevant government agencies and other systems like poultry production, while also ensuring the key opinions from attendees at our initial workshop are represented through inclusion of a subset of delegates from the fish farming stakeholder community. It is by this approach that acceptable, sustainable interventions to reduce ABU, and thus the problem of bacterial ABR, can be introduced to deliver beneficial change in the aquaculture sector.

A further limitation of this study is that participants and respondents lack of knowledge or experience with specific interventions may have influenced their responses to the survey. Though some information was provided in the descriptions of each intervention in the questionnaire, this may still have been insufficient for responders to make an informed judgement. This could potentially skew responses both ways, depending on how optimistic or pessimistic the responder may be towards a new intervention. This could be mitigated in future studies by providing more opportunities for training and discussion to achieve a shared understanding of the interventions prior to collecting participants' views. Language may represent a further barrier to understanding and, though reasonable steps were taken to mitigate this risk, it is not inconceivable that misunderstanding could have happened. The workshop was led in English by the research team, with concurrent translation into Arabic by a member of the research team fluent in both languages. Each workshop group was facilitated in English with a translator present. The survey was written in English and translated into Arabic before dissemination, whilst responses provided in Arabic were translated back into English by a member of our research team.

The range of suggested interventions above can be enacted by various stakeholders in the tilapia production system in Egypt. The benefit of the systems-thinking approach is that it allows for consequences of actions to be assessed and predicted, while also providing a means to select points in the system where the effectiveness of interventions could be evaluated through the collection of empirical data, thus helping to focus limited resources most effectively (Adam and de Savigny, 2012; Peters, 2014). These data can be used to confirm or quantify risks and could consist of measures such as concentration levels of antibiotic residues or presence of ARGs in the bacterial populations in the system.

In conclusion, this present study provides a foundation for the perceived acceptability and feasibility of possible interventions that can reduce ABU in tilapia production in Egypt and other countries with similar production systems and thus mitigate against the broader problems posed by ABR. This will help the sector to remain productive and increase its resilience to ABR but also to infectious diseases, while securing livelihoods and ensuring farmed tilapia is a safe and nutritious source of food. Systems-thinking and participatory approaches to create systems maps are important when determining the points within systems where interventions can be implemented, with feasibility and stakeholder acceptance being crucial considerations for the success of any intervention (Siokou et al., 2014). These same system maps can be used to assist in selecting points to monitor and assess the effectiveness of proposed interventions, a better understanding of which is crucial if we are to be successful in reducing overall ABU and tackling the global problem of ABR (Wernli et al., 2020).

## Ethical approval

Ethics approval for this work was granted by the Social Science Research Ethical Review Board at the Royal Veterinary College, under reference number: URN SR2020-0249.

## Funding

This work was supported by the 10.13039/501100000265UK Medical Research Council [award number MR/R015104/1]. C. V. Mohan and Shimaa Ali were supported by the CGIAR Research Program on Fish Agri-Food Systems (FISH) led by WorldFish. David C. Little and Mahmoud Eltholth were partially supported by BOLTI project (216429216 10.13039/100010897British Council UK Newton Fund, Institutional Links).

## Declaration of Competing Interest

The authors declare that they have no known competing financial interests or personal relationships that could have appeared to influence the work reported in this paper.

## Appendix A Supplementary data

Supplementary material Image 1
