# Supplementary material for: Systems-thinking approach to identify and assess feasibility of potential interventions to reduce antibiotic use in tilapia farming in Egypt
Source: Aquaculture. 2021 Jul 15;540:736735. doi: 10.1016/j.aquaculture.2021.736735 (PMC8164158; doi:10.1016/j.aquaculture.2021.736735)
Supplement: Supplementary file 1 — Supplementary material [file mmc1.pdf]

Systems-thinking approach to identify and assess feasibility of potential interventions to reduce antibiotic use in tilapia farming in Egypt

Supplementary material

S1: List of alternatives and interventions considered in post-workshop survey

**1**	**Improved farm management**<br>*This may relate to pond construction, fish stocking density, regular vet/ consultant fish health visits, proper waste disposal.*	
a	Pond construction	
b	Fertilisers	*Appropriate use and application*
c	Water	*Avoid use of agricultural drainage*<br>*Perform regular water exchange practices*
d	Feed	*Appropriate storage, protected from pests and vermin, and from spoilage and fungus*<br>*Use of high quality ingredients*<br>*Supplier of feed known to follow good hygienic and manufacturing practices*
**2**	**Improved genetics/breeds**	
a	Use of transgenic fish	
b	Selective breeding of fish	*Breeding of species/fish lines with traits known to be more resistant to infectious diseases.*
**3**	**Alternative therapies/products**	
a	Improved immunity	*Application of immunostimulants*<br>*Vaccination – water bath vaccination to improve fish health*<br>*Use of vitamin C*
b	Use of probiotics	
c	Phage therapy	*The use of bacteriophage (viruses that infect bacteria to treat bacterial diseases)*
d	Nanomaterials	*Materials with antibacterial properties. They may be engineered, incidental by-products or natural products, and have a size in at least one dimension of between 1 and 100 nanometres.*
e	Phytotherapy	*The use of natural plant based extracts. Some products are already available.*
**4**	**Diagnostic tools**<br>*This would allow investigation and identification of fish pathogens and diseases circulating in fish populations and would inform the selection of appropriate antibiotics to be used, therefore improving the chance of therapeutic cure, protecting fish health and productivity.*	
a	Rapid Diagnostic tests	
b	Routine use of antibiotic sensitivity testing	
**5**	**Monitoring and control**	
a	Water parameters	*Transparency, oxygen, etc.*
b	Microorganisms in the water	
c	Microorganisms in fish	
d	Data syndromes	*Based on observation of fish symptoms and behaviour*
**6**	**Biosecurity**	
a	Individual biosecurity	*Sourcing of spat from hatcheries with known high health status/ reputation for high quality spat. Control on the presence of other livestock species in the compound*
b	Collective biosecurity	*All farms that are connected would need to improve biosecurity. It relies on people's perceptions of biosecurity, and trusting that it works.*
**7**	**Rewards and incentives**	
a	Certification and quality assurance programs	
b	Producers associations, cooperatives	*Support from professional networks*
c	Expansion of trade market for exports	

8	**Education and training**	
a	Awareness raising	*Use of media, publicity and information programs - on the ABR risks for productivity and public health*
b	Training workshops	*One day workshops on disease prevention and control, best management practices, etc.*

S2: Questions posed to survey respondents

1 What type of organisation do you work for primarily?

- Academia
- Public sector
- Feed company
- Consultant
- Research institute
- Aqua shops
- Producer
- Other (please specify)

Then for each alternative or intervention listed above:

2 Feasibility – how feasible is this measure/intervention?

- Short term – it could be achieved in the next year
- Long term – it could be achieved in the next 10 years
- Not possible to achieve
- I don't know

3 Advantages

- Affordable
- Available in the market
- Easy to apply, conduct or implement
- Quick to conduct or implement
- I think that it would be effective
- I don't know

4 Disadvantages

- Expensive
- Not available
- Difficult to conduct or implement
- Time-consuming
- Lack of evidence about effectiveness – I need more evidence
- I don't know

5 Please rank the following type of interventions in order of importance to reduce the use of AB and improve aquatic health.

- Improved farm management
- Improved genetics / breeds
- Alternative therapies / products
- Diagnostic tools
- Monitoring and control
- Biosecurity
- Rewards and incentives
- Education and training
